# Utility of a next generation framework for assessment of genomic damage: A case study using the industrial chemical benzene

**DOI:** 10.1002/em.22346

**Published:** 2019-11-27

**Authors:** Mirjam Luijten, Nicholas S. Ball, Kerry L. Dearfield, B. Bhaskar Gollapudi, George E. Johnson, Federica Madia, Lauren Peel, Stefan Pfuhler, Raja S. Settivari, Wouter ter Burg, Paul A. White, Jan van Benthem

**Affiliations:** ^1^ Centre for Health Protection National Institute for Public Health and the Environment (RIVM) Bilthoven The Netherlands; ^2^ The Dow Chemical Company Horgen Switzerland; ^3^ Burke Virginia; ^4^ Center for Health Sciences Exponent, Inc. Alexandria Virginia; ^5^ Swansea University Medical School, Swansea University Swansea United Kingdom; ^6^ European Commission, Joint Research Centre (JRC) Ispra Italy; ^7^ Health and Environmental Sciences Institute Washington District of Columbia; ^8^ Procter & Gamble Mason Ohio; ^9^ Corteva Agriscience Newark Delaware; ^10^ Centre for Safety of Substances and Products National Institute for Public Health and the Environment (RIVM) Bilthoven The Netherlands; ^11^ Department of Biology University of Ottawa Ottawa Ontario Canada

**Keywords:** human health risk assessment, genotoxicity, mutagenicity, exposure, testing strategy

## Abstract

We recently published a next generation framework for assessing the risk of genomic damage via exposure to chemical substances. The framework entails a systematic approach with the aim to quantify risk levels for substances that induce genomic damage contributing to human adverse health outcomes. Here, we evaluated the utility of the framework for assessing the risk for industrial chemicals, using the case of benzene. Benzene is a well‐studied substance that is generally considered a genotoxic carcinogen and is known to cause leukemia. The case study limits its focus on occupational and general population health as it relates to benzene exposure. Using the framework as guidance, available data on benzene considered relevant for assessment of genetic damage were collected. Based on these data, we were able to conduct quantitative analyses for relevant data sets to estimate acceptable exposure levels and to characterize the risk of genetic damage. Key observations include the need for robust exposure assessments, the importance of information on toxicokinetic properties, and the benefits of cheminformatics. The framework points to the need for further improvement on understanding of the mechanism(s) of action involved, which would also provide support for the use of targeted tests rather than a prescribed set of assays. Overall, this case study demonstrates the utility of the next generation framework to quantitatively model human risk on the basis of genetic damage, thereby enabling a new, innovative risk assessment concept. Environ. Mol. Mutagen. 61:94–113, 2020. © 2019 The Authors. *Environmental and Molecular Mutagenesis* published by Wiley Periodicals, Inc. on behalf of Environmental Mutagen Society.

## INTRODUCTION

The testing of industrial chemicals for genetic damage has long been a focus of regulatory examination (e.g., Dearfield et al. 1991; Cimino 2006; EC 2008a; Eastmond et al. 2009; Ji et al. 2017). The testing is usually tiered: The first tier consists of a battery of genetic toxicity tests; any positive result generally triggers in vivo testing on the same genotoxicity endpoint. An in vivo positive result may lead to additional testing. The conclusion for any genetic damage usually relies on a yes/no binary decision (hazard identification) and does not examine the mechanism for the damage induced or characterize the risk the damage may pose to humans under exposure conditions.

Recent attention has focused on a paradigm shift that is pushing to innovate chemical risk assessment, and new concepts on how to achieve “next generation risk assessment” have been proposed (NRC 2007; Krewski et al. 2010; Embry et al. 2014; Pastoor et al. 2014). These concepts put more weight on exposure considerations compared to current approaches and aim for integration of sophisticated, preferably nonanimal, methods for hazard characterization, using increased understanding of mechanisms underlying toxicity as a starting point. Furthermore, it has been recognized that a wider range of genomic damage, including epigenetic alterations, is involved in many adverse health outcomes. More detailed understanding of mechanisms of genomic damage can play a key part in understanding disease progression and/or susceptibility, because genomic damage is now coupled to health issues and diseases beyond cancer. Further, better mechanistic understanding and assessment of genomic damage beyond a hazard identification approach may allow identification of levels below which exposure to a chemical poses negligible risk for genomic damage, that is, a “point of departure” (PoD) on the dose–response curve that can be utilized for risk assessment and management. Thus, new approaches that help unravel a broader range of mechanisms of action leading to genomic damage are key to enable these more information‐rich risk assessment concepts.

The Health and Environmental Sciences Institute (HESI) recently published a next generation framework for genomic damage (Dearfield et al. 2017). The framework, developed by HESI's Genetic Toxicology Technical Committee, is a flexible and therefore, at least in principle, widely applicable approach for assessing the risk of genomic damage via exposure to chemical substances. The purpose of the framework is to contribute to putting concepts for next generation risk assessment into practice. It puts greater emphasis on quantitative analyses and PoD determination with a focus on risk of humans under exposure conditions. Additionally, the approach aims to incorporate genomic, and not only genetic, damage into the assessments of risk. Hence, the approach is aimed at collecting information required to determine the risk posed by genomic damage, including estimates of risk.

The utility of the approach presented in Dearfield et al. (2017) for industrial chemicals was evaluated, using benzene as a case. The objective was to determine how the accumulated data (traditional genetic toxicity studies and other toxicity studies) could have been used following the more flexible framework to better assess the genotoxic hazard, characterize the risk of genetic damage, and possibly identify a PoD from any genetic damage observed. Thus, the emphasis of the case study was on the utility of the framework, and explicitly not to produce another detailed review manuscript on benzene. As this is the first time the framework is evaluated, we chose to limit the case study to genetic damage and not yet broaden the scope to genomic damage.

Benzene is a naturally occurring substance. During combustion processes, wood fires, and volcano eruptions, increased concentrations of benzene in ambient air can be measured. Benzene has been produced by humans since the 1800s from coal tar and later from petroleum. It also occurs naturally as a component of petroleum and of condensate from natural gas production, which is why many petroleum products contain residues of benzene. To date, benzene is still one of the most produced substances worldwide. Benzene is used primarily as a synthesis intermediate in the chemical and pharmaceutical industries for manufacturing of organic chemicals, such as styrene and cumene, and as an intermediate in the production of drugs, dyes, coatings, adhesives, insecticides, and plastics (ATSDR 2007, 2015; DECOS 2014; Eastmond et al. 2014). It is also added to gasoline for its octane‐enhancing and anti‐knocking properties (IARC 2012). Benzene was extensively used in industrial, professional, and consumer products as an organic solvent but was replaced over the years by less toxic solvents to meet occupational regulatory standards (Weisel 2010). Occupational human exposure to benzene has been associated with a range of acute and long‐term adverse health effects and diseases, including acute myeloid leukemia (Schnatter et al. 2005; Galbraith et al. 2010; Khalade et al. 2010) and myelodysplastic syndrome (Hayes et al. 1997; Schnatter et al. 2012; Collins et al. 2015; Copley et al. 2017; Li and Schnatter 2018). Benzene‐exposed individuals may also exhibit other adverse health effects such as hematotoxicity, which arises via multiple mechanisms and may be particularly evident among genetically susceptible subpopulations (Paustenbach et al. 1993; Snyder et al. 1993; Lan et al. 2004; Wang et al. 2012).

## BENZENE AS A CASE STUDY

The framework outlined in Dearfield et al. (2017) details a systematic examination of the reason(s) for genotoxicity testing, that is, what is known about the chemical under scrutiny and which data gaps need to be filled by testing to conclude on the genotoxicity potential. In brief, the framework comprises nine steps; see Table 1. The first step is planning and scoping, with the aim to clarify the problem and how it may be addressed. This is followed by the development of a knowledge base by gathering the existing information. After having created the biological argument, additional studies (if required) are identified and performed to test that biological rationale. Review of all the data leads to identification of the mechanisms of genetic damage involved as well as the dose at which the damage of concern is induced. The risk of causing damage at a given dose could then be established using actual (measured) or projected exposure data.

**Table 1:** Framework for Next Generation Risk Assessment

Step No.	Process	Benzene as case
1	Planning and scoping (incl. anticipated exposure)	Identify the relevant regulations in place for benzene Determine the most likely exposure route for benzene Determine the population group(s) of concern Determine the category of anticipated exposure
2	Determine expected exposure	Identify potential sources of benzene exposure for appropriate exposure route Determine expected pattern of exposure Estimate the level of benzene exposure for the population group(s) of concern
3	Build knowledge base	Collect information on ADME Collect information on physic‐chemical characteristics Chemoinformatics: generate data using QSAR software tools; include predictions on possible metabolites Collect available data from relevant in vitro and in vivo toxicity studies Collect data for other types of toxicity than genetic damage Collect mechanistic information
4	Create rational biological argument	Based on the knowledge gathered determine the potential of benzene for induction of genomic damage. If so, determine the most likely mechanism underlying this potential
5	Select assays and perform them	*Not applicable here as using published studies for benzene*
6	Review results	*Not applicable here as using published studies for benzene*
7	Select appropriate point of departure	Based on the rational biological argument identify relevant data set(s) Conduct quantitative analyses to derive a PoD
8	Estimate acceptable levels for endpoints of human relevance	Determine whether it is appropriate to use a nonlinear approach Using the derived PoD determine the acceptable level of daily exposure for the population group(s) of concern
9	Risk characterization	Estimate the risk for humans by applying MOE approach and by comparing exposure level to the acceptable level of daily exposure

Based on the framework described in Dearfield et al. (2017).

The purpose of the benzene case study was to evaluate the utility of the framework. More specifically, using the wide range of data available for benzene, we aimed to identify which information is key, unnecessary, or possibly misleading for decision making. For this, we conducted a retrospective analysis by using available data relevant for assessment of the potential of benzene to cause genotoxicity; Table 1 lists how the framework was applied. As benzene is a case study to evaluate the framework, we chose to mainly rely on data presented in review documents, such as those published by the Health Council of the Netherlands (DECOS 2014) and the European Chemicals Agency Committee for Risk Assessment (ECHA 2018). The data collected were reviewed to evaluate whether the framework led to the information needed for assessing the genotoxicity and eventually regulatory decision making. Studies in humans, such as in workers in the petrochemical industry, are obviously highly useful in the context of human health risk assessment. Nevertheless, available data from human studies were not considered here because this type of study would normally not be available for a new substance under evaluation.

### Planning and Scoping (Including Anticipated Exposure)

The main goal of the planning and scoping process is to establish the reason(s) for testing, that is, to define the purpose and scope of a risk assessment and the depth of the analysis (USEPA 2000, 2014; NRC 2009; Solomon et al. 2016; UN 2017). Relevant regulation(s) must be considered if an assessment is required for regulatory purposes. An important part of the planning and scoping step is the problem formulation, the systematic process to guide and direct what scientific questions need to be addressed in the risk assessment for the chemical under study. While planning and scoping outlines the broader questions to be addressed (including logistics, costs, etc.), problem formulation focuses on the more specific scientific questions regarding the chemical's potential to cause genomic damage relevant for human risk.

For benzene, an assessment of its genotoxic risk is considered an important contribution toward the risk management goal of protecting public health, assuming the assessment would produce the information required for decision making. In Europe, such a safety assessment performed by the producers is mandatory for classified substances manufactured or imported over 10 tonnes per annum because of the Registration, Evaluation, Authorisation and Restriction of Chemicals (REACH) regulation laid down in European Commission regulation no. 440/2008 (EC 2008a). In the United States, the Environmental Protection Agency (U.S. EPA) is required to evaluate both new and existing industrial chemicals and mixtures for their health and environmental risks under the Toxic Substances Control Act (TSCA) (USEPA 2019). New chemicals are subjected to premanufacturing notification/significant new use rules with higher regulatory scrutiny (TSCA Section 5) and, where necessary for existing chemicals, additional testing may be mandated to adequately assess their risks (TSCA Section 4). Other legislations require assessment of substances for specific situations but do not require information themselves. These situations include, for example, classification and labeling, transport, storage conditions, and evacuations in case of accidents. As these legislations do not require information, the availability depends upon the information requirements of the general legislation for industrial chemicals. Therefore, the information provided under the general industrial chemical legislation should also be sufficient to allow a risk assessment for specific situations. Given the use of benzene as a synthesis intermediate in various industrial processes and as a fuel additive, workers as well as the general population are of most concern, with inhalation as the most likely exposure route. It should be noted that benzene being a volatile substance may pose challenges to the assessment of its hazardous properties, especially regarding in vitro test systems.

In the context of this case study, we focused on both population groups, that is, on workers in various industries (e.g., petrochemical and manufacturing industries), and on the general population. According to the framework, categorization of anticipated exposure is proposed, ranging from minimal to high exposure [see Dearfield et al. 2017 for details], to define an initial base set of genetic toxicity testing assays. This base set may vary by category of exposure and size of population exposed. Benzene would fall into the “minimal/low exposure” category for workers in most industrialized developed countries because benzene is used primarily as a synthesis intermediate in (chemical) production plants and at oil refineries. Furthermore, protective measures have significantly improved over the last several decades, at least in Europe and Northern America. The potential for broad exposure category is applicable for the general population, because of widespread exposure due to the use of cigarettes, fueling at petrol stations, and motor vehicle emissions.

### Exposure Assessment

The human exposure assessment defines the target population and potentially susceptible individuals, as described later. It also defines the most important exposure sources, pathways, and routes. The assessment will identify the magnitude of cumulative exposures based on their source/activity, frequency, and duration. Relevance of the exposure assessment for decision making will then depend on the exposure profile and toxicological profile (including kinetics) of the substance (Geraets et al. 2016; Haber et al. 2016). An exposure assessment provides an estimate as a daily external or internal dose (using basic toxicokinetic data), an air concentration over a specified duration, or a dermal load. Exposures from several sources should be taken into account in the final so‐called aggregate exposure assessment.

Workplace exposure to benzene, where environmental concentrations of benzene are higher than in the general environment, occurs in Europe and Northern America mainly at oil refineries, drilling stations, and fueling stations, while this also involves other types of industry (eg, manufacturing) in other regions of the world (ATSDR 2007, 2015; Weisel 2010; Park et al. 2015). In addition to the background level, peak exposures may occur during maintenance and control, leaks, or other incidental releases. The frequency of occurrence of those peaks and the longest period without peaks is relevant for a risk assessment. Inhalation exposure is the predominant route for workers, although direct skin contact with certain high‐grade oils may be a relevant source of systemic exposure. Long‐term exposure to relatively high levels of benzene, compared to the low background level found in the general population, has been observed for workers (Weisel 2010). In Europe, long‐term occupational exposure to benzene is usually in the range of 0.05 ppm (0.16 mg/m^3^) to 0.1 ppm (0.3 mg/m^3^), due to its classification as a human carcinogen including a requirement to substitute benzene or minimize worker exposure. However, higher exposures have been reported for several tasks in the petrochemical industry (ECHA 2018). It should be noted that the worker situation in the European Union may not be representative of exposures in other (developed) countries. In China, exposures to benzene in the shoe manufacturing industry may even exceed levels of 100 ppm, according to a literature review (Wang et al. 2006; Weisel 2010).

The general population is exposed daily to relatively low concentrations of benzene resulting from industrial emissions and vehicle exhaust. In the European Union, the maximum content of benzene in gasoline, added as an anti‐knocking agent, was limited in 1998 to 1% (v/v) [EU Directive 98/70/EC relating to the quality of petrol and diesel fuels; EC 1998]. Subsequently, benzene concentrations in urban areas have decreased. ECHA reports several studies covering the 1990s to approximately 2015 where both urban and rural areas across Europe have been monitored. Air concentrations can vary but are within the range of <0.5 to 50 μg benzene/m^3^. To protect human health, a limit value of 5 μg benzene/m^3^ (1.5 ppb) has been set to improve air quality in the European Union (Directive 2008/50/EC (EC 2008b)); this limit value might still be exceeded in some urban areas (ECHA 2018). In other countries, exposures to benzene may be higher. Additional exposures result from cigarette smoking or passive smoking and refueling of vehicles (Wallace 1989; ATSDR 2007, 2015; ECHA 2018). Exposure to benzene in consumer products (substances and mixtures) and toys is restricted and is no longer considered a significant contributor within the European Union. Because cigarette smoke is a large contributor to benzene exposure, it is worthwhile to consider smokers separately from nonsmokers. Environmental tobacco smoke (passive smoking) can be a significant source of benzene exposure for nonsmokers (Wallace 1989). Arnold et al. (2013) state that smoking accounts for about 90% of cigarette smokers’ exposure to benzene. Gordon et al. (2002) state that benzene concentrations in exhaled breath can be 10–20 times higher for cigarette smokers, indicating that smokers experience peak exposures every time they smoke a cigarette. The benzene concentration in air inside a closed test chamber was elevated from a background level of 5 to 18 μg/m^3^ benzene after two cigarettes were smoked. Burning incense increased levels from 5 to 12, and even up to 205 μg/m^3^ benzene (Tirler and Settimo 2015).

### Absorption, Distribution, Metabolism, and Elimination Characteristics

Information on each of the absorption, distribution, metabolism, and elimination (ADME) components of toxicokinetics is highly relevant to both exposure and hazard assessment. Benzene is known to be readily absorbed by all physiological routes, including inhalation. Studies of the absorption of benzene after inhalation exposure have been published showing mean absorption rates ranging from approximately 50 to 80% (DECOS 2014). Data from case studies suggest that benzene, upon absorption, is readily distributed throughout the body. Benzene has been detected in various biological fluids and tissues of humans and has been shown to cross the human placenta. As benzene is lipophilic, the highest levels have been detected in lipid‐rich tissues (DECOS 2014). Animal studies show similar findings and also indicate that benzene distribution may depend on the perfusion rate of tissues, with higher levels being found in tissues with high perfusion rates, such as the kidney, lung, liver, brain, and spleen (DECOS 2014).

The metabolism of benzene occurs predominantly in the liver but also in the lung, with secondary metabolism occurring in the bone marrow (McHale et al. 2012). In brief, the metabolism of benzene starts with oxidation to benzene oxide mainly by cytochrome P450 2E1 (CYP2E1). Benzene oxide spontaneously rearranges to phenol, which subsequently results in catechol and/or hydroquinone metabolites, both of which can be additionally converted into toxic metabolites (Meek and Klaunig 2010). Alternatively, benzene oxide may be further metabolized to benzene dihydrodiol, which can be converted to catechol (Meek and Klaunig 2010; DECOS 2014). There is also the potential for a ring opening of the benzene leading to the formation of aldehyde metabolites. Inactivation occurs for example by an enzymatic reaction with glutathione. Some of the metabolites accumulate in the bone marrow where myeloperoxidases and other heme‐protein peroxidases further activate phenolic metabolites to semiquinone radicals producing reactive oxygen species, which inflict oxidative damage in various stem and progenitor cells and bone marrow niches (Snyder and Hedli 1996; Tuo et al. 1996; Hartwig 2010; McHale et al. 2012).

In the context of risk assessment, the complexity of benzene's metabolic properties is further increased due to genetic polymorphisms that have been reported for enzymes involved in benzene metabolism. These polymorphisms, including CYP2E1, glutathione‐*S*‐transferases GSTM1, GSTT1, and GSTP1, and NAD(P)H:quinone oxidoreductase 1, may influence both the toxification and detoxification of benzene (Carrieri et al. 2018; ECHA 2018). At least some of the reported polymorphisms seem to be associated with a higher susceptibility of developing leukemia (ECHA 2018). Furthermore, Carbonari et al. (2016) have shown that variability in polymorphic gene frequencies exists within and between Caucasian, Asian, and African populations, especially regarding GSTT1, GSTM1, and GSTA1.

Exhalation is the main route for excretion of unmetabolized benzene, whereas metabolized benzene is excreted primarily via urine (ATSDR 2007; ECHA 2018). Studies in humans and animals indicate that both exhalation and urinary excretion occur in several phases, with half‐lives of minutes to hours (ATSDR 2007). The half‐life for the slow phase of benzene elimination suggests an accumulation of benzene (ATSDR 2007).

### Hazard Assessment

#### In Silico Data

Computer‐assisted (in silico) quantitative structure–activity relationships (QSARs) and read‐across/analog identification methods provide predictions of the potential toxicity of a substance and are commonly used to aid initial assessment of hazard potential and prioritization for testing. Their use as tools to address toxicity endpoints (as part of a weight‐of‐evidence approach) for regulatory decision making is increasing. The framework points to these tools as excellent resources to relatively quickly provide insight to possible toxicities in the initial examination of chemicals as one builds the knowledge base for assessment. Examples include predicting QSARs of bacterial mutagenicity for impurities in pharmaceuticals, as defined in International Conference on Harmonisation of Technical Requirements for Registration of Pharmaceuticals for Human Use Guideline M7 (ICH 2014) and under the European REACH regulation supporting the use of read‐across, or determining whether full or limited registration is needed for low‐tonnage substances. These recent developments have fueled investment and improvements in (Q)SAR and read‐across approaches, which are both based on structural similarity and/or the presence or absence of molecular features that give alerts for a certain toxic property of a substance. In that context, it is hypothesized that a substance with similar key structural features to another chemical for which sufficient toxicity data are available will also act via the same mechanism of action and have similar toxicity potential. It is important to note that this concept is usually applied only if a substance has no actual testing data for a given endpoint but could provide important input into a weight‐of‐evidence assessment to resolve questionable or conflicting data and generates insights into potential mode(s) of action.

There are numerous studies available for benzene that provide a comprehensive assessment of its genotoxicity. Consequently, including (Q)SAR and/or read‐across methods in decision making may therefore not be an obvious choice. However, in the context of the evaluation of the utility of our next generation framework (Dearfield et al. 2017), in silico information is seen as an important building block to provide insight into the potential for genotoxic mechanisms. We performed a (Q)SAR and read‐across evaluation for benzene and its relevant analogs with a focus on genotoxicity endpoints. First, potential analogs of benzene were identified using both Organization for Economic Co‐operation and Development (OECD) Toolbox models and the analog search strategy as described by Wu et al. (2010). The chemicals identified were then rated (Wu et al. 2010), resulting in five chemicals classified as “suitable with precondition” (phenol, catechol, hydroquinone, benzene oxide, and 1,2‐dihydro‐1,2‐dihydroxybenzene; Table 2), all of which are metabolites of benzene. The precondition implies that the metabolite does form in vivo, and thus internal (systemic) exposure to these metabolites can be expected to occur. Next, benzene and all suitable with precondition analogs were subjected to a detailed QSAR analysis using Derek Nexus (version 6.0.1, 2018; Lhasa Limited, Leeds, UK) and OASIS Times (version 2.29.1.88; Laboratory of Mathematical Chemistry, Bourgas, Bulgaria) software. It should be noted that benzene data were not part of the training sets used for the development of these tools. An overview of the predictions obtained with the two software packages is provided in Table 3, which also lists the relevance of the prediction according to OASIS Times. Neither benzene nor any of the five analogs themselves triggered an alert for mutagenicity in bacteria or clastogenicity in in vitro tests in these software tools. However, the tools indicated an alert for clastogenicity in in vitro tests for metabolites of three analogs (benzene oxide, catechol, hydroquinone), and an alert for mutagenicity in bacteria for metabolites of benzene oxide. Besides indicating potential for genetic damage, these results suggest that the metabolism involved is complex. Also, the alerts provide insight to which type of genetic tests (clastogenicity here) would be useful in targeting testing for potential genotoxic activity (i.e., more focused testing vs. a standard battery approach).

**Table 2:** Overview of Analogs With a “Suitable With Precondition” Rating for Benzene

Structure	CAS RN	Chemical name	Analog rating	Description
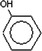	108–95‐2	Phenol	Suitable with precondition	Predominant urinary metabolite of benzene
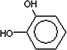	120‐80‐9	Catechol	Suitable with precondition	Metabolite of benzene
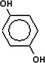	123‐31‐9	Hydroquinone	Suitable with precondition	Metabolite of benzene
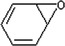	1488‐25‐1	Benzene oxide	Suitable with precondition	Epoxidation metabolite of benzene
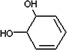	75453‐80‐4	1,2‐Dihydro‐1,2‐dihydroxybenzene	Suitable with precondition	Metabolite of benzene

**Table 3:** Prediction of the Genotoxicity Potential of Benzene and Its Suitable Analogs Using Derek Nexus and OASIS TIMES Software

	Benzene			Benzene oxide			Phenol		
SMILES	c1ccccc1			C1=CC=CC2C1O2			Oc1ccccc1		
Structure	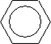			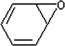			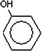		
Endpoints	DEREK	OASIS		DEREK	OASIS		DEREK	OASIS	
Software version	Derek Nexus v.6.0.1	TIMES v.2.29.1.88	Relevance	Derek Nexus v.6.0.1	TIMES v.2.29.1.88	Relevance	Derek Nexus v.6.0.1	TIMES v.2.29.1.88	Relevance
Ames mutagenicity^a^	Inactive	Negative	Relevant	Plausible	Parent (−), metabolites (+)	Relevant	Inactive	Parent (−), metabolites (+)	Disregard
Chromosome damage (in vitro)^b^	No alert	Negative	Uncertain	Plausible	Parent (−), metabolites (+)	Uncertain	No alert	Parent (−), metabolites (+)	Uncertain
Nonspecific genotoxicity (in vitro)	No alert	Not available	n.a.	No alert	Not available	n.a.	No alert	Not available	n.a.

	1,2‐Dihydro‐1,2‐dihydroxybenzene			Catechol			Hydroquinone		
SMILES	OC1C=CC=CC1O			Oc1ccccc1O			Oc1ccc(O)cc1		
Structure	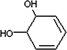			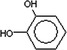			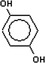		
Endpoints	DEREK	OASIS		DEREK	OASIS		DEREK	OASIS	
Software version	Derek Nexus v.6.0.1	TIMES v.2.29.1.88	Relevance	Derek Nexus v.6.0.1	TIMES v.2.29.1.88	Relevance	Derek Nexus v.6.0.1	TIMES v.2.29.1.88	Relevance
Ames mutagenicity	Inactive	Parent (−), metabolite (+)	Relevant	Inactive	Parent (−), metabolite (+)	Disregard	Inactive	Parent (−), metabolite (+)	Disregard
Chromosome damage (in vitro)	No alert	Parent (−) metabolite (+)	Uncertain	Probable (exact example match)	Parent (−), metabolite (+)	Uncertain	Plausible	Parent (−), metabolite (+)	Uncertain
Nonspecific genotoxicity (in vitro)	No alert	Not available	n.a.	No alert	Not available	n.a.	No alert	Not available	n.a.

n.a., not applicable.

^a^For Ames mutagenicity, Derek specifies that there are “no misclassified or unclassified features” in the benzene molecule.

^b^For chromosome damage in vitro, the OASIS call “uncertain relevance” corresponds to <70% similarity with successful AND < 5% similarity with unsuccessful predictions.

#### In Vitro Data

A wealth of information on in vitro testing of genetic damage induced by benzene or its metabolites is available. It should be noted that benzene is a volatile substance, which generally is challenging to test appropriately in an in vitro test system. Additionally, the use of S9 mix may be inadequate to mimic in vitro the metabolic capacity required for the complex metabolism of benzene. Therefore, we chose not to collect data only for benzene itself, but also for a limited set of metabolites. In light of the purpose of the present case study, we focused on benzene and the five metabolites that were predicted by in silico tools to be of relevance for human exposure.

As shown in Table 4, benzene administration induced micronuclei (MN) in a Chinese hamster lung (CHL)/IU cell line (Matsushima et al. 1999) as well as in MCL‐5 cells; however, no response was detected in human lymphoblast AHH‐1 cells (Crofton‐Sleigh et al. 1993). In an interlaboratory validation study using V79 cells with one treatment and one sampling time only, benzene was found to produce negative results after three hours of exposure with metabolic activation and a 21‐hour recovery period (von der Hude et al. 2000). It was argued that the use of dimethylsulfoxide as solvent, which reduces the metabolism of benzene, and the short recovery period used in the test were factors responsible for the negative response. On the other hand, induction of MN was observed in a genetically engineered V79‐derived cell line expressing both human CYP2E1 and human sulfotransferase SULT1A1 (Jiang et al. 2014), confirming the key role of CYP2E1 and Phase II enzymes in the metabolism of benzene and production of active metabolites.

**Table 4:** Summary of in vitro Genotoxicity Findings for Benzene and Its Metabolites

Substance	Micronucleus		Chromosomal Aberrations and SCE		Ames		Gene Mutation in Mammalian Cells	
	Overall call	Results	Overall call	Results	Overall call	Results	Overall call	Results
Benzene	[+]	[**−**] AHH‐1 cells; [+] CHL(IU) and MCL‐5 cells; [**−**]V79 cells, three hour exposure, 21 hr sampling	[+]	[+] human lymphocytes; [+] CHO cells; [+] CHL cells; [+] SHE cells, aneuploidy	**[−]**	[−] TA97, TA98, TA100, TA1535 w/out S9; [E] TA97 with S9; [−] TA102 with CYP2E1; [+] TA1535 but not dose‐dependent with S9	[E]	*Tk*: [+] lymphoma cells; *Hprt*: generally [**−**]; [+] only one study
Phenol	[+]	[+] CHO cells w/out S9; [+] V79 cells without S9; weak [+] human lymphocytes without S9	[I]	n.t.	[−]	[**−**]TA97, TA98, TA100, TA102, TA104, TA1535, w/out S9 (many studies)	[+]	*Tk*: weak [+]; *Hprt*: weak [+] V79 cells; [**−**] V79 cells but at 24 hr treatment; [+] SHE cells
Catechol	[+]/[−]	[+] V79 cells; [+] human lymphocytes; [**−**] weak increase in human lymphocytes	[+]	[+] SHE cells, aneuploidy; [+] in CHO cells; [+] SCE (several studies)	[−]	[**−**]TA97, TA98, TA1535, TA1537, TA2637, TA102, TA104, w/out S9	[+]	*Tk*: [+] lymphoma cells; *Hprt*: [+] SHE cells; [+] V79 cells
Hydroquinone	[+]	[+] CHL(IU) cells	[+]	[+] CA at <10 μg/mL, possible aneuploidy; [+] SCE	[−]	[**−**] TA97, TA98, TA100, TA1535, TA137 w/out S9	[+]	*Tk*: [+] lymphoma cells
Benzene oxide	[I]	n.t.		n.t.	[I]	[**−**] TA98, TA100 w/out S9; [+] TA100; [**−**] TA1535 with S9; [+] TA98, TA100 with S9		n.t.
1,2‐dihydro‐1,2‐dihydroxy‐benzene	**[−]**	Weak response in V79 cells	**[−]**	No clear evidence of SCE in V79 cells	**[−]**	[**−**] TA98 w/out S9; [**−**] TA100, TM677/8 AZAG. w/out S9; [+] TA100, TM677/8 AZAG. with S9; weak [+] TA98; [+] TA104; [+] TA1535 with S9, desiccator	[+]	Weak increase gene mutations (6‐thioguanine resistance) in V79 cells

Data are from Glatt et al. (1989), Whysner et al. (2004), Stark and Rastetter (1996), Kirkland et al. (2016), and Toxicology Data Network (2019). Overall calls and data results are presented for *in vitro* genotoxicity findings of benzene and its metabolites. CHL, Chinese hamster lung; CHO, Chinese hamster ovary; [E], equivocal overall call is given if result is questionable or inconsistent within a study, if a positive response cannot be dismissed, or if both positive and negative findings across different studies show apparent equal validity; [I], inconclusive overall call is given in the case of negative or unclear results, where no firm conclusion can be made in terms of meeting the requirements of the current OECD Test Guidelines or recommended best practices; n.t., not tested; SCE, sister chromatid exchange; SHE, Syrian Hamster Embryo; w/out S9: tested with and without S9 metabolic activation.

Data on chromosome aberrations and sister chromatid exchange (SCE) are also summarized in Table 4. For the chromosome aberration test in vitro, an overall negative result was reported in the U.S. National Toxicology Program (NTP) database after exposure to 16–1000 μg/mL, while there was positive evidence for SCE in Chinese hamster ovary (CHO) cells in the absence of S9 (Gulati et al. 1989). A clastogenic response to benzene has been observed in human lymphocytes, which also showed a significant increase of aneuploidy in the absence of rat liver S9 (Ishidate Jr. et al. 1988). When metabolic activation systems were included, benzene has been reported positive in other types of cells as well, including CHL and CHO cells (Ishidate Jr. 1985; Ishidate Jr. et al. 1988). Numerical aberrations were confirmed in other studies showing aneuploidy in the near diploid range of Syrian hamster embryo cells (Tsutsui et al. 1997). A significant dose‐dependent increase in cells with chromosomal aberrations was observed more recently in cultured bovine peripheral lymphocytes (Sivikova et al. 2005).

In the Ames test, benzene has been consistently classified as a nonmutagen (Table 4). Within the range of 1.5–1000 μg/plate, benzene was tested in standard strains TA97, TA98, TA100, and TA1535 with and without metabolic activation using both the Aroclor‐induced male Sprague–Dawley rat liver S9 mix and the male Syrian hamster liver S9 mix. Despite a slight toxicity at the highest concentration tested and an increase (although not dose‐dependent) in revertants in TA97, the overall outcome in the NTP database is negative (NTP 2019). A number of other studies and reviews reported similar conclusions (Baker and Bonin 1985; Rexroat and Probst 1985; Zeiger and Haworth 1985; Brams et al. 1987; Jung et al. 1992; Muller et al. 1993; Kirkland et al. 2005, 2011). The nonmutagenicity of benzene in bacteria has been linked to the inadequacy of the S9 microsomal activation system (Yardley‐Jones et al. 1991). In fact, it was reported that Aroclor was not able to induce the P450 enzymes involved in the biotransformation of benzene into active mutagenic products. However, Burke et al. (1994) reported negative results also in TA102 in the presence of inducers of CYP2E1. Additional metabolic pathways in bacteria were then suggested. A positive, but not dose‐dependent, result was reported only in TA1535 in the presence of NADPH‐fortified post‐mitochondrial fractions (S9 mix) from rat and mouse liver homogenates using a protocol with a desiccator (Glatt et al. 1989).

In gene mutation tests in mammalian cells, benzene has been repeatedly observed to induce gene forward mutations in the L5178Y *Tk* ^+/−^ mouse lymphoma assay (MLA) (Oberly et al. 1985; Styles et al. 1985; Oglesby et al. 1989; Sofuni et al. 1996; Mitchell et al. 1997; Kirkland et al. 2005, 2011). Instead, no increase of such mutations was reported in the NTP database (NTP 2019). By contrast, results obtained for the *Hprt* locus were predominantly negative, as reported by many authors (Amacher and Turner 1985; Fox and Delow 1985; Kuroda 1985; Zdzienicka and Simons 1985; Oberly et al. 1990). Only Tsutsui et al. (1997) reported positive results for the *Hprt* locus. The overall positive findings in the MLA and overall negative *Hprt* data support a predominantly clastogenic effect of benzene, as the induction of small colonies due to slow growing mutants in the MLA is considered indicative of clastogenicity rather than a point mutation (OECD 2015). On the other hand, other experts considered the results for benzene uninterpretable upon review (Kirkland et al. 2011; Schisler et al. 2018).

Taken together, we concluded that benzene has the potential to induce clastogenic and possibly aneugenic effects, while the evidence for its potential to induce gene mutations is poor (Table 4). This conclusion is based on the data presented and not on all studies available; however, it is in line with a recent assessment of ECHA's Risk Assessment Committee (RAC) (ECHA 2018). Remarkably, benzene induced chromosome aberrations in the presence of S9 in mammalian cells, while in bacteria the lack of response was attributed to inadequacy of S9. This highlights the need to explore relevant in vitro data for metabolites of benzene. Evaluation of in vitro genotoxicity test results for the five metabolites of benzene (see the “In Silico Data” section and Table 2) revealed that these metabolites, like the parent compound, do not induce a strong mutagenic response in bacteria, except for some evidence in specific strains, as in the case of 1,2‐dihydro‐1,2‐dihydroxybenzene (Table 4). Other in vitro genotoxicity data (besides mutagenicity in bacteria) were not available for benzene oxide. Positive findings have been reported for genotoxicity in mammalian cells for the remaining four metabolites: phenol, catechol, hydroquinone, and 1,2‐dihydro‐1,2‐dihydroxybenzene. The available data suggest a clastogenic potential, as MN, chromosomal aberrations, and SCEs have been observed at different dose levels and conditions [Whysner et al. 2004, summarized in Table 4]. These results do not imply automatic inclusion of the assessment of genotoxic potential for all metabolites. However, in cases where metabolism is suspected to be complex, additional data on metabolites may aid in detailed understanding of the mechanisms involved, like here for benzene and its metabolites. The chemical nature, rate of formation, presence of limiting enzymes, and metabolic pathways to which they belong in the various tissues may explain the diversity of responses and potency observed across the different metabolites and may contribute to the genotoxicity effects of the parent compound (Glatt et al. 1989; Whysner et al. 2004; Kirkland et al. 2016).

#### In Vivo Testing

A large number of in vivo genotoxicity studies have been conducted for benzene. As inhalation is considered the most relevant route of exposure for this case study, we focused on in vivo studies using this route of exposure. The resulting findings are summarized in Table 5. In transgenic rodent studies, exposure to benzene via inhalation resulted only in marginal (<twofold) and inconsistent increases in mutant frequency in the lung and spleen (Mullin et al. 1995, 1998); however, a larger increase in mutant frequency was observed in splenic T‐cells in mice after long exposures (up to 38 weeks) to high doses (Albertini et al. 2010). Inhalation of benzene induced increased levels of DNA damage in peripheral blood, bone marrow, and liver at different doses in the in vivo comet assay in mice (Plappert et al. 1994). Findings from in vivo micronucleus tests (MNTs) showed a consistent response: each of the studies identified showed an increase in MN in bone marrow in mice (wild type and genetically modified) and rats (Erexson et al. 1986; Luke et al. 1988; Farris et al. 1996; Healy et al. 2001; French et al. 2015). In mice, increases in MN were also observed for peripheral blood (Tice et al. 1989; Farris et al. 1996; French et al. 2015). The positive results in the MNT were found after both short‐ and long‐term (up to 24 weeks) exposure to doses up to 1,000 ppm (Table 5). The magnitude of the response is dependent on the duration and frequency of exposure as well as on the sex and strain of mice. The MN response in male mice was greater compared to females, consistent with benzene‐mediated higher carcinogenic responses in males (Luke et al. 1988; Tice et al. 1989). It has been suggested that this gender difference in sensitivity is (partly) due to hormonal factors (Siou et al. 1981).

**Table 5:** Summary of in vivo Genotoxicity Findings for Benzene

Species/Strain	Exposure regimen	Result	Reference
**Gene mutation**			
B6C3F1 transgenic lambda/lacI mice	300 ppm; 6 hr/day, 5 days/week, 12 weeks	Marginal increase in mutant frequency in lung and spleen at 300 ppm	Mullin et al. (1995, 1998)
C57BL/6 p53^+/−^ and C57BL/6 WT mice	100–200 ppm; 6 hr/day, 5 days/week, 38 weeks	Increase in mutant frequency in splenic T‐cells at 100 ppm	Albertini et al. (2010)
**DNA damage (comet)**			
BDFI mice	100–900 ppm; 6 hr/day, 5 days/week, six weeks	Positive in liver, peripheral blood, and bone marrow at 100 ppm	Plappert et al. (1994)
**Chromosome aberration**			
DO mice	1–100 ppm; 6 hr/day, 5 days/week, 4 weeks	MN: positive in bone marrow and peripheral blood at 1 ppm	French et al. (2015)
DBA/2 mice	10–1000 ppm; six hours	MN: positive in bone marrow at 10 ppm	Erexson et al. (1986)
Sprague–Dawley rats	0.1–30 ppm; six hours	MN: positive in bone marrow at 1 ppm	Erexson et al. (1986)
Tg.p53^+/−^ and Tg.AC mice and wild‐type counterparts (FVB/N and C57BL/6)	100–200 ppm; 6 hr/day, 5 days/week, 38 weeks	MN: positive in bone marrow at 100 ppm	Healy et al. (2001)
B6C3F1 mice	1–200 ppm; 6 hr/day, 5 days/week, for one, two, four, or eight weeks	MN: positive in bone marrow and peripheral blood at 100 ppm	Farris et al. (1996)
DBA/2 mice	300 ppm, 13 weeks	MN: positive in bone marrow at 300 ppm	Luke et al. (1988)
B6C3F1, DBA/2, and C57BL/6 mice	300 ppm, 5 days/week, 13 weeks	MN: positive in peripheral blood at 300 ppm	Tice et al. (1989)

DO, diversity outbred; MN, micronuclei.

Rodent studies focused on DNA reactivity (measured using ^32^P‐postlabeling or DNA‐binding studies) did not suggest preferential binding of benzene or its metabolites with rat or mice DNA in target tissues showing neoplasia. At dose levels producing neoplasia in rodents, benzene or its metabolites did not cause adducts in the Zymbal gland (neoplastic target organ for benzene), liver, kidney, mammary gland, or bone marrow (Whysner et al. 2004, and references therein).

Various studies providing relevant information to better understand the mechanisms underlying the toxicity of benzene were identified. Studies in *Cyp2e1* ^−/−^ mice demonstrated once more the key role of the CYP2E1 enzyme in benzene‐mediated toxicity, as *Cyp2e1* ^−/−^ mice did not exhibit genotoxicity or cytotoxicity in the blood, bone marrow, thymus, and spleen, in contrast to wild‐type mice (Valentine et al. 1996). The aryl hydrocarbon receptor (AhR) participates in CYP2E1 induction and thus in benzene‐mediated toxicity, as no hematotoxicity was induced in *AhR* ^*−/−*^ mice (Yoon et al. 2003). Benzene was shown to inhibit topoisomerase II enzyme activity in an isolated enzyme system, in a human bone marrow‐derived leukemia cell line, and in vivo in the bone marrow of treated mice via oral gavage (Eastmond et al. 2001). The latter in vivo study also demonstrated that benzene induces both chromosome breakage and aneuploidy, with chromosome breakage being the predominant effect, while aneuploidy was a relatively infrequent event (Eastmond et al. 2001). Inhibition of topoisomerase II enzyme activity was also observed in in vitro studies testing metabolites of benzene (Hutt and Kalf 1996; Lindsey Jr. et al. 2004; Eastmond et al. 2005). The hypothesis that the genotoxic effects of benzene are secondary to oxidative stress and inhibition of topoisomerase II was further substantiated by mechanism‐oriented transcriptomics studies in p53‐knockout mice and wild‐type mice (Yoon et al. 2003; Faiola et al. 2004).

Overall, the in vivo tests for chromosome aberrations confirmed the positive findings observed under in vitro conditions. All chromosome aberrations and MNTs evaluated for this case study were positive. Additionally, the in vivo data provided relevant information regarding characteristics of benzene and the mechanisms involved in its genotoxic potential, including its capacity to induce aneuploidy. The results obtained from the in vivo gene mutation tests are in line with the poor evidence for benzene's potential to induce gene mutations in vitro: The data collected suggest this potential is very weak and is only detected when using large cumulative doses (time × concentration). This is consistent with increasing evidence that gene mutations can also be induced as a consequence of primary toxicity (eg, inflammatory processes) (Wickliffe et al. 2016) rather than direct DNA reactivity. Consequently, the weight‐of‐evidence of available data for benzene supports a conclusion that benzene is predominantly a clastogenic as well as aneugenic substance.

#### Other Relevant Toxicity Data

Like for any other toxicological effect, the potential/potency of a substance to induce genetic toxicity or genomic damage is not evaluated in isolation. Information obtained from toxicity studies targeting other endpoints can be very useful to better understand the mode(s) of action involved, in particular when these appear to be fairly complex. Ideally, when available, human studies should be carefully evaluated during a risk assessment. However, as mentioned before, data from human studies were not considered in this benzene case study because this type of study would normally not be available for a new substance under evaluation.

For benzene, information obtained from repeated‐dose toxicity studies and reproductive toxicity studies in experimental animals is considered relevant when assessing genomic damage. These animal studies have shown that benzene has the potential to induce toxicity in the hematological system, including significantly reduced counts of erythrocytes, leukocytes, and platelets and other evidence of adverse effects on blood‐forming units (ATSDR 2007; ECHA 2018). Adverse immunological effects in response to inhalation exposure of benzene have also been observed in animal studies. The effects include damage to both humoral (antibody) and cellular (leukocyte) responses (ATSDR 2007), which was shown in animal studies to result in impaired cellular immunity (ECHA 2018). Regarding reproductive toxicity, effects on fertility and development have been observed in animals only at very high doses (ECHA 2018).

### Mode of Action

Epidemiological data on benzene link high levels of benzene exposure, among other effects, to various forms of leukemias and cytogenetic effects (ECHA 2018). The data collected in the previous sections inform that the metabolism of benzene is inherently complex and results in the formation of numerous reactive and toxic metabolites as well as reactive oxygen species. Benzene has the ability to induce chromosomal damage, which seems a plausible mode of action (MOA) for its carcinogenicity through interaction of its metabolites with macromolecules of target cells. Benzene has weak potential to induce gene mutations and primary DNA reactivity of benzene and/or its metabolites seems of little importance in relation to carcinogenesis. The genotoxic effects of benzene are suggested to be secondary to oxidative stress and inhibition of topoisomerase II (Yoon et al. 2003; Faiola et al. 2004), that is, nonlinear mechanisms. Benzene and/or its metabolites are also known to alter cell proliferation (Meek and Klaunig 2010) and thus provide an environment for the proliferation of genetically initiated cells, eventually leading to tumors. This abridged MOA based on the genetic toxicity data alone is consistent with that proposed by Meek and Klaunig (2010) taking into consideration other known biological effects of benzene. The latest International Agency for Research on Cancer monograph (IARC 2018) concluded that benzene, in addition to being genotoxic, exhibits many of the key characteristics of carcinogens as described by Smith et al. (2016). These include its ability to (1) become electrophilic upon metabolic activation, (2) alter DNA repair processes, (3) induce genomic instability, (4) alter the epigenome, (5) suppress the immune system, (6) induce oxidative stress, (7) alter cell proliferation, and (8) modulate receptor‐mediated effects, in particular the AhR. This is supported by studies investigating the mechanisms by which benzene affects hematopoietic stem cells to cause toxicity and cancer (McHale et al. 2012; Wang et al. 2012; Li et al. 2018). Thus, there is growing evidence that benzene is acting via multiple pathways in eliciting its tumorigenic response, although most of the key characteristics listed above are not independent of each other. An alternate explanation might be that a clear understanding on its MOA is still elusive. Despite the many mechanisms benzene can influence, the quantitative analyses in the next session focuses, for illustration of the framework, on genotoxicity only.

### Quantitative Analyses

One of the goals of the framework is to quantify the risk of substances that induce genomic damage on the basis of experimental data. Given that we consider benzene a predominantly clastogenic and aneugenic substance, data on MN induction were considered appropriate for quantitative analyses to assess genotoxicity. Again, such a quantitative analysis should ideally be conducted using data from human studies. However, human studies often vary greatly in quality, for example, with regard to accuracy of exposure assessment and control for confounding factors (ECHA 2018). Moreover, this type of study would normally not be available for a new substance to be evaluated. Therefore, for the purpose of this case study we chose to use MN data from a data‐rich NTP/National Institute of Environmental Health Sciences study conducted in Diversity Outbred (DO) mice (French et al. 2015). Although this study is not fully compliant with OECD Test Guideline 474 (OECD 2016), especially in terms of genotype and number of animals used per dosing group, we consider this study useful to illustrate how quantitative analysis of data on genetic damage could be employed for risk assessment.

**Figure 1:**
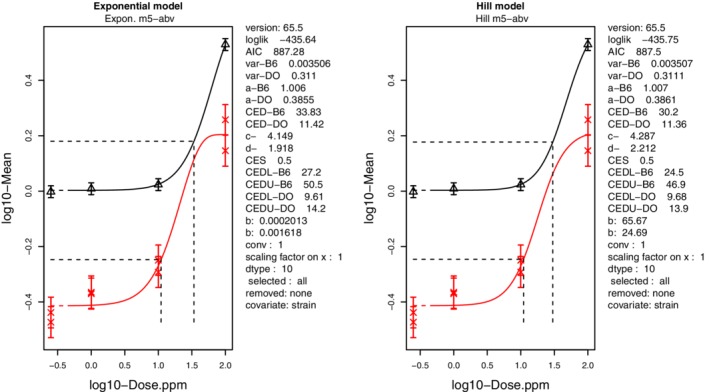
Benzene genotoxic potency values determined for DO and B6C3F1 mice using BMD analyses with strain as covariate. The response (Y‐axis) metric is MN frequency in bone marrow‐derived reticulocytes in %, and the X‐axis metric is airborne benzene concentration in ppm. Data were fitted with the exponential continuous data model (left panel) and the Hill model (right panel) using a CES of 50%. The data shown in black indicate the B6C3F1 response; red shows the DO response. Values shown are means with confidence intervals as standard deviation. The text adjacent to each panel provide curve fit parameters (i.e., AIC and log likelihood), mean background for each levels of the covariate (i.e., parameter a), BMD values (i.e., CED or Critical Effect Dose), estimated maximum response and log steepness (i.e., parameters c and d), and BMD (CED) confidence lower and upper confidence limits.

DO mice are genetically heterozygous and carry a complex mixture of alleles; hence, these mice have been suggested to mimic interindividual variation in humans (French et al. 2015). The study involved benzene exposure (0, 1, 10, or 100 ppm) of male DO mice (75 animals/exposure group) via inhalation for 28 days (six hours/day for five days/week). Measurements of chromosomal damage included MN frequencies in reticulocytes (RETs) from bone marrow. We analyzed these data using the advanced combined covariate benchmark dose (BMD) approach, which generally improves the precision in the estimated BMDs (Slob 2002; Slob and Setzer 2014). This approach requires multiple data sets that are comparable with respect to the endpoint, tissue, and route of exposure; thus, dose–response data from a similar experiment in B6C3F1 mice were also used (Farris et al. 1996). Quantitative analysis using the BMD approach also requires specification of a predefined fractional increase in response above the concurrent control, a value commonly referred to as the benchmark response (BMR) or critical effect size (CES). For continuous data, the EFSA recommended default value for a CES (BMR) is 5%, but the effect size “may be modified based on statistical or toxicological considerations” (EFSA 2009). For the quantitative analysis of data on genotoxicity, which is a recent development, initially a CES of 10% was used (MacGregor et al. 2015a, 2015b; Wills et al. 2016a, 2016b; Long et al. 2018). However, two subsequent analyses have shown independently that a CES value of 50% would be more appropriate for genetic toxicity data such as those obtained from the in vivo MN test (Slob 2017; Zeller et al. 2017). Therefore, combined covariate BMD analysis of the two data sets was performed using a CES of 50% at which the response constitutes an increase deemed appropriate for this endpoint. To note, unlike tumor data, genotoxicity data are considered continuous data like, for example, body weight. This analysis, which was performed in PROAST (version 65.5; http://www.proast.nl) with strain as the covariate, resulted in a BMC_50_ of 11.4 ppm benzene for DO mice, with 9.6 ppm and 14.2 ppm as the lower (BMCL_50_) and upper (BMCU_50_) confidence limits, respectively (Fig. 1). In a risk assessment, this BMC_50_, including its confidence limits, can be used as a PoD for assessing human risk associated with genomic damage.

### Estimate Acceptable Levels for Endpoints of Human Relevance

The next step in the framework is to estimate the acceptable level of a daily exposure to the human population (including vulnerable subgroups) that is likely to be without an appreciable risk of deleterious effects during a lifetime (USEPA 2015). Focusing on the genotoxic damage (the observed clastogenic and aneugenic effects) by benzene that is considered secondary in nature (see the “Mode of Action” section), a traditional risk assessment using assessment factors can be applied to determine the acceptable level of daily exposure as described later (DECOS 2014; AGS 2016; ECHA 2018).

An acceptable level of a daily exposure to the human population is determined by extrapolating from the PoD of the key experimental data set by correcting for uncertainty through the use of assessment factors (AFs). While the AFs applied do vary across regulatory jurisdictions and geographical regions, they conceptually agree in that the combined AFs are thought to account for all (various) sources of uncertainty. Typically, a composite or total AF of 100 is derived by multiplying a factor of 10 for animal to human extrapolation (interspecies) and a factor of 10 for variability within human populations (intraspecies). These AFs can be adjusted based on various considerations, including species differences and allometric scaling or differences in absorption, distribution, metabolism, and pharmacokinetics. Additional AFs are sometimes used to account for differences in the duration and/or frequency of exposure, severity of toxicity endpoint, and uncertainty in the PoD (MacGregor et al. 2015a). It is important to mention that the use of AFs to extrapolate from genotoxicity data is a new area and there is ongoing discussion in the expert community but no agreement to date as to what AFs to use in this context. The use of AFs serves the purpose of illustrating how this can work in practice but should not be seen as setting a precedent in this respect. We highly encourage further discussion in this area.

For the benzene case study, the first step is to correct the PoD for differences between the exposure regimen used for the animal study and the likely human exposure scenario. There are multiple approaches to achieve this; here, we performed the same calculations as done by RAC (ECHA 2018). DO mice were exposed for six hours/day, whereas workers are assumed to be exposed for eight hours/day. Furthermore, workers are assumed to have a relatively enhanced breathing pattern compared to animals because of a higher level of activity. This leads to a theoretical increase in the volume of inhaled air over eight hours from a human‐equivalent volume of 6.7 m^3^ to 10 m^3^ (ECHA 2012). Hence, the BMCL_50_ and BMDU_50_ values of 9.6 ppm and 14.2 ppm, respectively, derived from the DO mouse study would translate to a human‐equivalent PoD range of 4.8 ppm to 7.1 ppm (i.e., animal PoD * (6/8 * 6.7/10)). As a next step, AFs would be applied to derive an acceptable range of a daily exposure to the human population. The AFs chosen to be applied in this case study include factors for intraspecies and interspecies extrapolation (interspecies variability in toxicokinetics and toxicodynamics), study duration, and effect severity. Even for endpoints that have a much longer history of use of risk quantification methods, there is no globally harmonized approach to which AFs (type and value) should be used; this depends on the nature of the regulatory purpose and the regulatory jurisdiction.

For illustrative purposes only, we have determined the acceptable range of a daily exposure to workers and to the general population in Europe. For workers, we applied the same AFs as used by RAC, where appropriate (ECHA 2018). According to the RAC opinion, appropriate AFs would include an AF of 2.5 to account for interspecies variability in toxicokinetics and toxicodynamics and an AF of 6 to extrapolate the findings from a 28‐day animal study to a chronic exposure. For intraspecies variability an AF of 5 would apply, plus an additional AF of 3 to account for the severity of the effect (ECHA 2018). In this example, application of a composite AF of 225 (2.5*6*5*3) to the human‐equivalent PoD range of 4.8–7.1 ppm would result in an acceptable range of a daily exposure of 21–32 ppb for workers. For the determination of an acceptable range of a daily exposure for the general population in Europe, we followed ECHA guidance (ECHA 2012). For this population group, exposure is not necessarily limited to working hours but may occur throughout the day; in a worst‐case scenario this could be 24 hr. On the other hand, the overall level of activity could be assumed to be similar between the general population and the animals in the respective study. Based on these assumptions, the BMCL_50_ and BMDU_50_ values of 9.6 ppm and 14.2 ppm derived from the DO mouse study would translate to a human‐equivalent PoD range of 2.4 ppm to 3.6 ppm (i.e., animal PoD * 6/24). The same AFs as used for workers can apply, except for the AF for intraspecies variability; an AF of 10 instead of 5 was considered more appropriate as a higher variability is expected in the general population versus industry workers. Together, this would result in a composite AF of 450 (2.5*6*10*3), leading to an acceptable range of a daily exposure of 5.3–8 ppb for the general population. These values for acceptable levels of a daily exposure are examples and may change depending on agreements of the expert community. For instance, there is currently no consensus regarding the need of an additional AF that accounts for effect severity. An AF of 3–10 would be consistent with recommendations by ECHA and by ICH for oncogenic effects (ECHA 2012, 2018; ICH 2016). Also, the AF used to account for study duration is based on the assumption that the effects observed after 28 days need adjustment when extrapolated to lifetime exposure, which may not be supportable for the endpoint used (clastogenicity and aneugenicity as measured in the micronucleus assay). Micronucleus formation is an acute response to the exposure that reaches a steady state (MacGregor et al. 1990) and exposure times beyond acute can be used but not required as defined in OECD guideline 474 and the assay has shown similar sensitivity for acute and subchronic exposures (Witt et al. 2000).

### Risk Characterization

To determine if humans are at risk, the dose at which a hazard (toxicity) occurs is compared with exposure estimates for every specific scenario. For this, as described in our framework for next generation risk assessment of genomic damage (Dearfield et al. 2017), one may apply the margin of exposure (MOE) approach that was developed by EFSA for substances that are both genotoxic and carcinogenic (EFSA 2005). As benzene's genotoxic activity is considered secondary in nature, we have not applied the MOE concept for benzene but focus on the outcome of the traditional risk assessment detailed in the previous section. In our example following the approach taken by ECHA's RAC, the acceptable range of a daily exposure to benzene was 21–32 ppb for workers, whereas the long‐term average exposure levels estimated for workplaces in Europe is in the range of 50–100 ppb (0.05–0.1 ppm). For the general population, we determined in our example an acceptable range of a daily exposure of 5.3–8 ppb for genetic damage. As laid out in the “Exposure Assessment” section, the limit value for air concentrations in the European Union is set at 5 μg benzene/m^3^ (1.5 ppb) for all effects of benzene (EC 2008b). It should be noted that the assumption that the public would inhale air containing this limit concentration of 1.5 ppb for 24 h a day, over a lifetime, is very conservative.

We would like to stress that the aforementioned examples for workers and the general population are a simplification, as reflected by using the limit value for air concentrations in the European Union as a daily exposure estimate for the general population. Additionally, other aspects such as availability of personal protection equipment and exposure from smoking and refueling were not considered. Daily exposure estimates would increase for smokers, as smoking is considered a substantial contributor to benzene exposure (Wallace 1989; Arnold et al. 2013). Thus, the examples are provided solely to illustrate how the framework could be applied as a contribution to a comprehensive risk assessment. In general, risk managers consider aspects such as the context of use, availability of personal protection equipment, the severity of the effect, the toxicological mechanisms involved, the number of assumptions used, and the size of the affected population when evaluating the risk. This will affect the risk management decision if the risks can be considered acceptable or need to be decreased, for example, by measures to reduce exposure.

## EVALUATION AND CONCLUSIONS

The purpose of this case study was to evaluate the utility of the framework we developed for assessing the risk of genomic damage due to exposure to chemical substances. Ideally, we would have applied the framework in a prospective approach where testing outcome and human health consequences were unknown. This is quite challenging for a data‐rich substance like benzene, and would have required additional measures such as a blinded setup of the case study. Therefore, we employed a retrospective approach for this case study.

To evaluate the framework, we reviewed all the information and data collected, taking into account the anticipated exposure category determined in the planning and scoping phase. The qualitative categorization of the likely exposure scenarios is intended as a screening tool for setting priorities (Dearfield et al. 2017). For workers, the anticipated exposure category was “minimal” (“Planning and Scoping” section). Therefore, it was concluded that a limited data set may be considered if focusing solely on workers: Predictions of human exposure levels for the most likely exposure scenario, cheminformatics (in silico predictions) indicating complex metabolic processes may be involved and informing on possible genotoxicity testing outcomes, in vitro genotoxicity testing providing alerts for clastogenicity and aneuploidy, and an in vivo MNT confirming clastogenic and possibly aneugenic effects. The latter would also serve as a source for deriving PoDs and acceptable levels, to allow for risk characterization. It should be noted that the in silico predictions for benzene and the five analogs themselves could be interpreted incorrectly as “negative,” if the indirect alerts stemming from reactive metabolites were not taken into account. Given that benzene is used primarily as a synthesis intermediate and measures are in place to protect workers from benzene exposure, extensive investigations to unravel all details of the mechanism(s) underlying cancer are not considered directly necessary if workers would have been the only population group of concern. It should be noted, however, that also possible incidents impacting production facilities and their immediate environment need to be included in a risk assessment. Furthermore, investments in understanding the mechanisms of toxicity involved are worthwhile, as these data can be reused for substances that have a similar MoA.

For the general population, the situation would be somewhat different because wide exposure was anticipated. Because of the probable increased concern due to the anticipated high number of exposed individuals, additional investigation into the relevance to human risk is called for in the framework. Therefore, more robust and appropriate exposure assessments and toxicity testing would likely be required. These may include more detailed exposure assessments (eg, addressing effects of nonexposed periods on toxicity and the influence of short‐term high exposures), leading to better insight into internal exposure levels. All of the data and information analyzed in this retrospective case study show what would likely have occurred in a prospective approach in a similar scenario. Furthermore, besides the information described earlier for the worker population, better insight into the ADME properties of benzene, including identification of a number of metabolites, some of which can react with macromolecules such as DNA, and more detailed understanding of the involved MOAs would have been required for a proper assessment of the genotoxic potential. Some understanding of the MOA would have been achieved with the information collected (in case a prospective approach was taken), but it is clear that this step in the framework is critical to highlight the need for possible additional actions/investigations to investigate genomic damage. In our view, although not discussed here in detail, the adverse outcome pathway (AOP) concept (Ankley et al. 2010; OECD 2013) could also be used to ensure better understanding of the mechanisms involved. This would also support the use of targeted tests rather than a prescribed set of assays as is customary in some regulatory settings. Increased understanding of biological pathways triggered by a chemical can provide mechanistic insights that support the understanding of the relevance of (genotoxic) effects to human risk assessment. Such enhanced understanding is also key for selecting key events (and thus studies) to be used for quantitative analyses and PoD determination. In the case of benzene, it seems plausible that multiple mechanisms (ie, a network of pathways) are involved in carcinogenesis. Delineating this network, including identification of key events and quantification of their relationships, would be highly valuable to determine whether a PoD based on genetic damage is the most appropriate key event or perhaps overly conservative.

The benzene case study confirmed once more that the outcome of a risk assessment is also driven by its purpose: The assessment for workers yielded different safety margins compared to the assessment done for the general population. In our example, this difference in safety margins was partly attributed to the anticipated larger variability within the general population. However, the same level of variability could be seen, by chance, in a worker population. Another factor explaining the difference in safety margins is the difference in the exposure estimates between the population groups, and it has to be emphasized that the assumption of a daily exposure at the defined upper limit of benzene content in ambient air over lifetime for the general population seems highly conservative. The large impact of the exposure estimation on the calculations underpins the need for robust exposure assessment which currently is often lacking.

Another element that should be addressed with sufficient care is toxicokinetics, because each of the ADME components is highly relevant for exposure estimation and risk assessment. For example, information on elimination indicated the accumulation of benzene, whereas knowledge on the complexity of benzene metabolism appeared to be essential for the interpretation of both in silico findings and test results from in vitro systems, as well as understanding of the mechanisms underlying genetic damage. Furthermore, insight into toxicokinetic properties contributes to proper assessment of interindividual variability in susceptibility of adverse health outcomes. The relevance of knowledge on metabolic processes involved was also apparent from the results obtained with cheminformatics, that is, the (Q)SARs and read‐across/analog identification methods. Besides the likelihood of a metabolite to be formed in vivo, the software tools used indicated alerts for in vitro clastogenicity of secondary metabolites of benzene. This nicely demonstrates the benefit of using these tools in an early phase of an assessment: Besides information on potential hazard, the predictions for benzene also point to careful consideration of metabolic competency of the in vitro tests to be used. These additional information requirements on exposure and toxicokinetics clearly exceed the current information requirements (at least for industrial chemicals in the EU), especially as the amount of available information for benzene is already much larger than for most other substances.

Finally, it was ascertained that application of the framework requires integration of different areas of expertise. Although this holds also true for the risk assessment approaches currently in place, it was considered increasingly relevant due to the flexible nature of the new approach. Consequently, intensified interdisciplinary collaborations may be required. A more flexible approach is expected to increase cost efficiency compared to traditional approaches to chemical hazard characterization. On the other hand, external and internal exposure assessments may increase the assessment cost. A drawback of the framework could be that it is more difficult to predict the total cost of hazard identification, exposure quantification, and risk assessment; however, this may be partly resolved by an accurate and detailed problem formulation.

Taken together, this case study provides proof‐of‐principle that the next generation framework for assessment of genomic damage is a useful contribution to a comprehensive risk assessment. The framework enabled a more efficient risk assessment process by providing all elements needed for decision making in a systematic manner. Also, the flexibility inherent in the approach fueled broader thinking and the generation of more information for a fuller understanding of potential human risk. Although there is a great amount of data on benzene available as can be seen in the retrospective analysis here, inserting this into the framework in a prospective way demonstrated the utility of this next generation framework. Additional case studies, both for other industrial chemicals and other types of products, would be useful to further refine the approach. Preferably, these future case studies would incorporate aspects of next generation risk assessment that were not (sufficiently) addressed in the present case study, such as application of the AOP concept to guide toxicity testing and the organization of mechanistic information, addressing genomic (including epigenetic, and not only genetic) damage, and have greater emphasis on the use of non‐animal methods for toxicity testing. Such case studies are expected to further improve the framework, which we believe has shown to be useful for the benzene case, thereby contributing to achieve next generation risk assessment.

## AUTHOR CONTRIBUTIONS

M.L. provided leadership to the GTTC “Clean Sheet” workgroup discussions and led the preparation and writing of the manuscript. N.S.B., K.L.D., B.B.G, G.E.J., F.M., S.P., R.S.S., W.T.B., P.A.W., and J.V.B. all provided written contributions pertaining to their expertise and participated in the preparation and editing of the manuscript. L.P. participated in the preparation and editing of the manuscript and provided logistical, organizational, and editorial support for the project. All authors approved the final manuscript.

## Conflict of Interest

Nicholas Ball is an employee of The Dow Chemical Company®, a company that produces products containing benzene. B. Gollapudi works for a consulting firm, which provides technical expertise to its client(s) on issues related to the toxicity of benzene.

## Disclaimer

This document represents the consensus of the authors’ views expressed as individual scientists, and does not necessarily represent the policies and procedures of their respective institutions.
